# Epidural hematoma after total hip arthroplasty in ankylosing spondylitis patient: A case report and review of the literature

**DOI:** 10.1097/MD.0000000000006859

**Published:** 2017-05-12

**Authors:** Jia Li, Ke Qi, Yongjin Zhang, Chenchen Xue, Weidong Xu

**Affiliations:** Department of Orthopedics, Changhai Hospital Affiliated to the Second Military Medical University, Shanghai, China.

**Keywords:** ankylosing spondylitis, epidural hematoma, total hip arthroplasty

## Abstract

**Rationale:**

Ankylosing spondylitis (AS) can affect the hip joint, causing deformity and disability. Total hip arthroplasty can obviously relieve the pain of the hip joint, and reconstruct the function of hip joint. Epidural anesthesia in this patient population has high risk of epidural hematoma, but the reason is unclear.

**Patient concerns:**

A 44-year-old man diagnosed with AS underwent total hip arthroplasty.

**Diagnoses:**

Six days after operation, he was diagnosed epidural hematoma.

**Interventions:**

Laminectomy and decompression surgery was performed.

**Outcomes:**

At the last follow-up, he recovered the feeling and function of lower limbs. A literature review was undertaken to understand the incidence and risk factors. The incidence of spinal hematoma in this population is high and only probable risk factors are reported without further research.

**Lessons:**

Based on our review and the illustration of this case, AS patients have both lumbar and hip bony fusion. The exact bone canal caused by the lumbar puncture needle may play an important role in epidural hematoma. General anesthesia may be a better choice for this special patient cohort.

## 1 Introduction

Ankylosing spondylitis (AS) is a chronic immune-mediated inflammatory disease characterized by inflammation that predominantly affects the axial skeleton. Literature has reported that 30% to 50% of patients with AS exhibited involvement of the hips, and among those with the hips affected, 90% presented bilaterally.^[1]^ Total hip arthroplasty (THA) can obviously relieve the pain of the hip joint, and reconstruct the function of hip joint.^[2]^

Anesthesia plays an important role in the course of surgery. Both general anesthesia and spinal anesthesia are chosen in THA. Rodgers et al^[3]^ had compared the effects of the 2 anesthesia styles by meta-analysis. In their study, 141 trials with 9559 patients were included. They found that spinal anesthesia had the benefit of reducing total mortality. Meanwhile, the risks of deep vein thrombosis and pulmonary embolism, which were high in THA, were also reduced in patients undergoing spinal anesthesia. They recommended more widespread use of spinal anesthesia, and this anesthesia style is also more suitable for patients with THA.

However, for AS patient groups, the lumbar spine is fused, and spinal anesthesia becomes very difficult. Taking into account the incidence of this pathology in the entire population, the incidence of spinal hematoma in this population is high.^[4–6]^ There are several probable risk factors, such as repeated lumbar puncture and nonsteroidal anti-inflammatory drug (NSAID) intake. However, no reports have focused on the exact bone canal caused by the lumbar puncture needle, which may play an important role in epidural hematoma.

Here, we present a case of epidural hematoma after THA for AS. The patient underwent a failed spinal anesthesia, and changed to general anesthesia. Five days after THA, there was a chronic epidural hematoma, the lower limbs were in complete paralysis, and the patient was incontinent. Through a timely posterior lumbar decompression surgery, he recovered.

## 2 Case report

**Figure 1:**
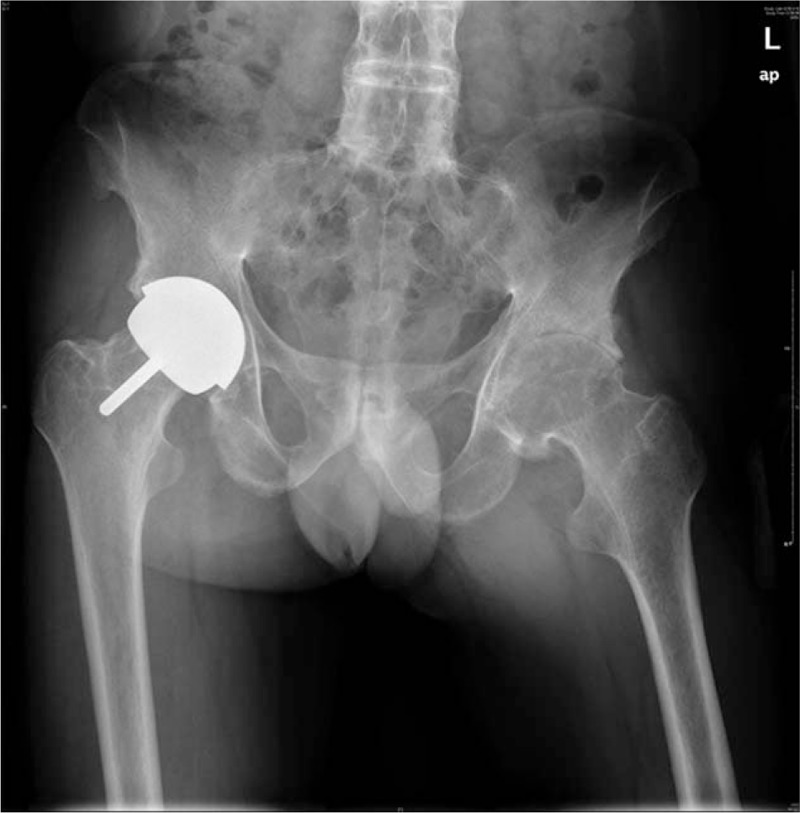
The radiograph of the pelvis of the patient shows bony ankylosis of the lumbar vertebra and bilateral sacroiliac joints. The right hip underwent hip resurfacing 5 years ago, and the left hip was narrowing.

A 44-year-old man diagnosed with AS was admitted to our clinic on April 24, 2016. He suffered with pain and bony ankylosis of both hips for 10 years. He had the diagnosis of AS for 20 years. Five years ago, he had a THA in his right hip, and now, he is ready to do the left side. The physical examination showed restricted joint movements. The range of motion of the left hip was: flexion 30 degrees, extension 0 degrees, adduction 10 degrees, abduction 20 degrees, and external rotation 20 degrees. Laboratory examinations were in normal range. The routine blood examination showed: hematocrit (HCT) 43.1%, hemoglobin (HGB) 143 g/L; coagulation function: prothrombin time (PT) = 14.1 seconds, activated partial thromboplastin time (APTT) = 38.8 seconds, thrombin time (TT) = 15.0 seconds, international normalized ratio (INR) = 1.1. The C-reactive protein (CRP) was 13.4 mg/L, and the erythrocyte sedimentation rate (ESR) was 16 mm/H. A preoperative X-ray showed that the left hip had bony ankylosis and the right side of the hip was replaced (Fig. 1).

After a preoperative examination, a THA was performed on April 25, 2016. The anesthesiologist attempted spinal anesthesia at the L2 to L3 intervertebral space, but failed. The anesthesiologist then changed to general anesthesia. The anesthesia and operation were successful. Because there was oozing of blood from the osteotomy surface and femoral medullary cavity, we ordered 400 mL red cell suspension and 200 mL plasma for transfusion. Tranexamic acid 1 g was injected intravenously before the incision was closed.

On the first day after the operation, the patient complained of pain in the spine, and could not lie down. After he turned to the lateral position under our guidance, the lumbar pain was slightly relieved. On the second day after the drainage was removed, he was asked to start functional recovery, with ankle pump exercise and quadriceps muscle contraction. Because AS patients have more bleeding, we delayed the initiation time of anticoagulation. The anticoagulation therapy was started on the third day, with rivaroxaban, 10 mg, qd. He suffered dysuria in the following days.

**Figure 2:**
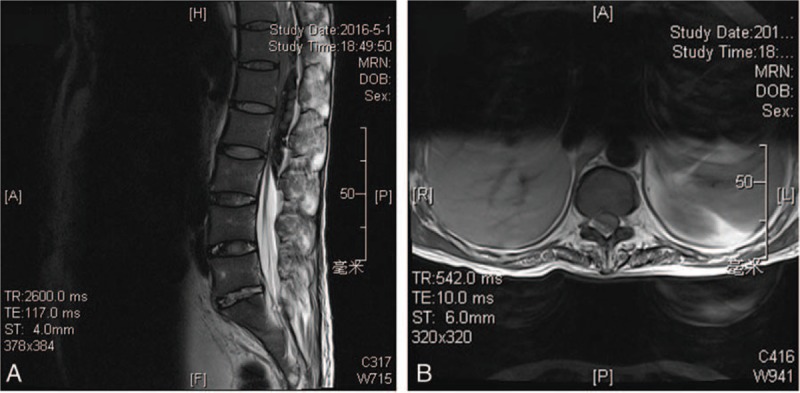
(A, B) MRI showed epidural hematoma was formed from T11 to L3 level and compression of the dural sac. (A) The sagittal image; (B) the cross-sectional image. MRI = magnetic resonance imaging.

On the sixth day in the morning, the patient suddenly complained of not being able to move his lower limbs, and the muscle strength was 0. We did a thoracic and lumbar magnetic resonance imaging (MRI) examination at once, and the result showed that an epidural hematoma had formed (Fig. 2). The routine blood examination showed: HCT 23.3%, HGB 76 g/L; coagulation function: PT = 13.2 seconds, APTT = 33.0 seconds, TT = 14.7 seconds, INR = 1.0. We ordered 400 mL red cell suspension and 400 mL plasma for transfusion. The next morning, a posterior laminectomy and a decompression of thoracolumbar was arranged, and another 800 mL red cell suspension and 400 mL plasma was ordered for transfusion.

The first day after the hematoma removal, the patient recovered stool sensation, but a catheter was still indwelled. The lower limbs’ muscle strength recovered to grade 3, and sensory disturbance level was down to mid-thigh level. The routine blood examination showed: HCT 29.3%, HGB 95 g/L; coagulation function: PT = 15.2 seconds, APTT = 28.2 seconds, TT = 15.8 seconds, INR = 1.2. The lumbar drainage tube was removed the next day, and hyperbaric oxygen therapy was started. Two months after the lumbar decompression operation, the feeling and function of the lower limbs were restored, and the muscle strength was restored to level 5, without incontinence.

## 3 Discussion

An epidural hematoma is a rare complication after spinal anesthesia. Wulf^[7]^ has reported 51 cases of epidural hematomas after lumbar anesthesia, 5 of which were AS patients. The author pointed out that AS was a new, previously unreported risk factor, whereas the reason for the epidural hematoma was unknown. The risk factors for epidural hematoma after spinal anesthesia may be: the higher incidence of difficult, traumatic attempts to identify the epidural space due to anatomical abnormalities, the pretreatment with analgesics such as NSAIDs, the higher incidence of epidural hematomas resulting in cord compression, and neurological symptoms due to a narrow epidural space with smaller foramina. However, the exact reason for this complication is still unknown.

In our case, the patient who was diagnosed with AS underwent THA. Spinal anesthesia was tentative, and when the patient complained of numbness on one side of the lower limb, the anesthesiologist changed to general anesthesia. There was no repeated puncture. The patient had stopped NSAIDs for a month, and the coagulation tests found no obvious abnormalities. We offered intraoperative tranexamic acid for hemostatic treatment, and the anticoagulation therapy was started on the third day. None of the rate factors above was included in this case.

**Figure 3:**
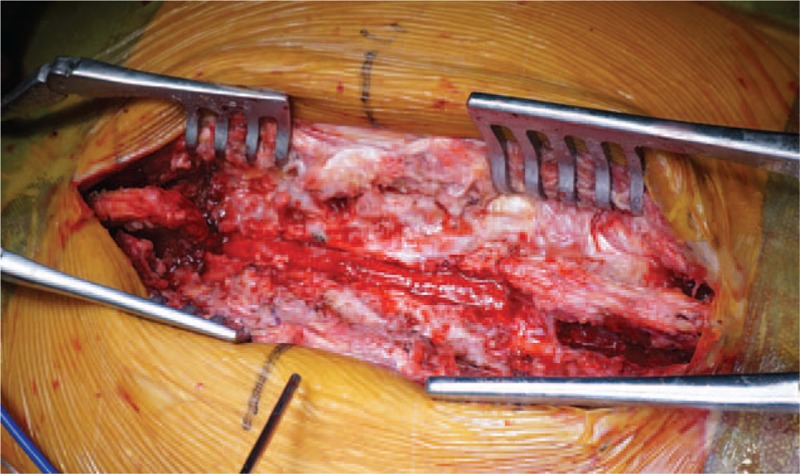
Thoracolumbar posterior decompression was finished. The decompression ranged from T11 to L3. Intraoperative exploration showed complete fusion of the vertebral, without any intervertebral space. After the lamina was resectioned by laminectomy rongeur, all the wound was sealed by bone wax, without active bleeding.

We have reported that bone surface bleeding is the main reason for blood loss for AS after THA.^[8]^ The blood volume was calculated according to the formula described by Nadler et al.^[9]^ Perioperative total blood loss was estimated based on the Hb balance method.^[10]^ Until the morning of the sixth day, the calculated total blood loss was 1491 mL, and the total hidden blood loss was 851 mL, partly from the surgical site, and the rest from the lumbar puncture site. The hematoma removed from the lumbar decompression can confirm the chronic bleeding from the puncture site, and this was the direct reason for incomplete paralysis after THA (Fig. 3).

Thoracic laminectomy decompression surgery itself has the risk of bleeding. The laminectomy intraoperative also has the risk of blood oozing in the bone surface. Therefore, on one hand, the bone surface needs to be sealed by bone wax; on the other hand, indwelling drainage was also needed before incision closure. After the content of the drain was stable and less than 20 mL, the drainage was removed.

## 4 Conclusions

Patients with AS have both lumbar and hip bony fusion. When treating them with surgical intervention, attention should be paid to the bone surface bleeding. In addition to anesthesia, these patients also have a great risk of forming an epidural hemorrhage after a lumbar puncture. If the patient is confirmed for lumbar bone fusion, general anesthesia is a better choice.
